# The organization of sleep–wake patterns around daily schedules in college students

**DOI:** 10.1093/sleep/zsad278

**Published:** 2023-11-01

**Authors:** Sinh Lu, Julia E Stone, Elizabeth B Klerman, Andrew W McHill, Laura K Barger, Rebecca Robbins, Dorothee Fischer, Akane Sano, Charles A Czeisler, Shantha M W Rajaratnam, Andrew J K Phillips

**Affiliations:** Turner Institute for Brain and Mental Health, School of Psychological Sciences, Monash University, Clayton, VIC, Australia; Turner Institute for Brain and Mental Health, School of Psychological Sciences, Monash University, Clayton, VIC, Australia; Department of Neurology, Massachusetts General Hospital, Boston, MA, USA; Division of Sleep and Circadian Disorders, Departments of Medicine and Neurology, Brigham and Women’s Hospital, Boston, MA, USA; Division of Sleep Medicine, Harvard Medical School, Boston, MA, USA; Division of Sleep and Circadian Disorders, Departments of Medicine and Neurology, Brigham and Women’s Hospital, Boston, MA, USA; Division of Sleep Medicine, Harvard Medical School, Boston, MA, USA; Sleep, Chronobiology, and Health Laboratory, School of Nursing, Oregon Health and Science University, Portland, OR, USA; Division of Sleep and Circadian Disorders, Departments of Medicine and Neurology, Brigham and Women’s Hospital, Boston, MA, USA; Division of Sleep Medicine, Harvard Medical School, Boston, MA, USA; Division of Sleep and Circadian Disorders, Departments of Medicine and Neurology, Brigham and Women’s Hospital, Boston, MA, USA; Division of Sleep Medicine, Harvard Medical School, Boston, MA, USA; Department of Sleep and Human Factors Research, Institute for Aerospace Medicine, German Aerospace Center, Cologne, Germany; Department of Electrical and Computer Engineering, Rice University, Houston, TX, USA; Affective Computing Group, Media Lab, Massachusetts Institute of Technology, Cambridge, MA, USA; Division of Sleep and Circadian Disorders, Departments of Medicine and Neurology, Brigham and Women’s Hospital, Boston, MA, USA; Division of Sleep Medicine, Harvard Medical School, Boston, MA, USA; Turner Institute for Brain and Mental Health, School of Psychological Sciences, Monash University, Clayton, VIC, Australia; Division of Sleep and Circadian Disorders, Departments of Medicine and Neurology, Brigham and Women’s Hospital, Boston, MA, USA; Division of Sleep Medicine, Harvard Medical School, Boston, MA, USA; Turner Institute for Brain and Mental Health, School of Psychological Sciences, Monash University, Clayton, VIC, Australia; Division of Sleep and Circadian Disorders, Departments of Medicine and Neurology, Brigham and Women’s Hospital, Boston, MA, USA; Division of Sleep Medicine, Harvard Medical School, Boston, MA, USA

**Keywords:** sleep timing, actigraphy, daily schedule, academic, exercise, extracurricular activities

## Abstract

The amount of time available in a day is fixed, and consequently, sleep is often sacrificed for waking activities. For college students, daily activities, comprised of scheduled classes, work, study, social, and other extracurricular events, are major contributors to insufficient and poor-quality sleep. We investigated the impact of daily schedules on sleep–wake timing in 223 undergraduate students (age: 18–27 years, 37% females) from a United States university, who were monitored for ~30 days. Sleep–wake timing and daily recorded activities (attendance at academic, studying, exercise-based, and/or extracurricular activities) were captured by a twice-daily internet-based diary. Wrist-worn actigraphy was conducted to confirm sleep–wake timing. Linear mixed models were used to quantify associations between daily schedule and sleep–wake timing at between-person and within-person levels. Later scheduled start time predicted later sleep onset (between and within: *p* < .001), longer sleep duration on the previous night (within: *p* < .001), and later wake time (between and within: *p* < .001). Later schedule end time predicted later sleep onset (within: *p* < .001) and shorter sleep duration that night (within: *p* < .001). For every 1 hour that activities extended beyond 10 pm, sleep onset was delayed by 15 minutes at the within-person level and 40 minutes at the between-person level, and sleep duration was shortened by 6 and 23 minutes, respectively. Increased daily documented total activity time predicted earlier wake (between and within: *p* < .001), later sleep onset that night (within: *p* < .05), and shorter sleep duration (within: *p* < .001). These results indicate that daily schedules are an important factor in sleep timing and duration in college students.

**Clinical Trial**: Multi-scale Modeling of Sleep Behaviors in Social Networks; **URL**: https://clinicaltrials.gov/study/NCT02846077; **Registration:**NCT02846077.

## Graphical Abstract

**Graphical Abstract:**
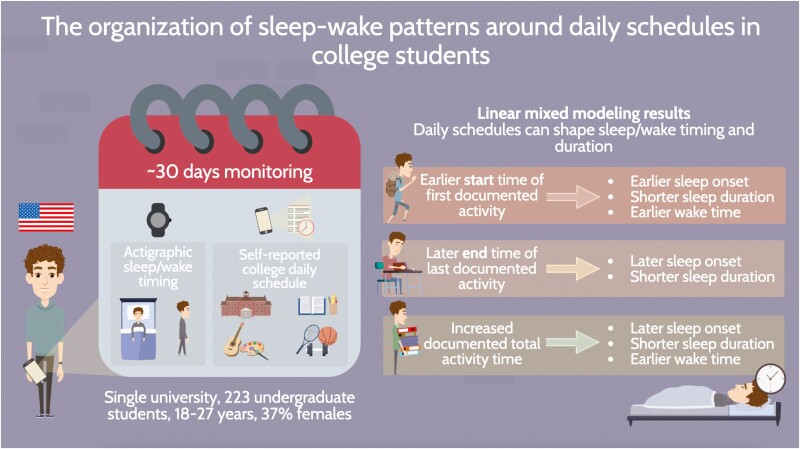

**Statement of Significance**

College students frequently experience insufficient and irregular sleep. A potential reason is the competing demands for time, including class work, exercise, socialization, and extracurricular activities. We investigated how different components of daily schedules predict the sleep–wake timing of college students. We found that acute changes in scheduled start time, scheduled end time, and daily documented total activity time were associated with notable changes in sleep–wake timing and duration. These findings indicate the importance of appropriate timing of daily events for shaping healthy sleep–wake patterns.

## Introduction

College students consistently sleep less than the recommended 7–9 hours per night, and report that their sleep is often irregular and of poor quality [1–4]. One potential contributor to these sleep issues is the variety of competing demands on time, including scheduled classes, exercise, socialization, and extracurricular activities [5–8]. While considerable attention has been given to the effects of environmental factors (e.g. excessive noise [9], technology or light exposure before bed [10], substance use [11], mental health disorders [12], and other social stressors [13, 14]) on sleep, the relationship between sleep and activity schedules on a daily basis remains relatively understudied. Understanding and improving sleep health among college students is crucial due to its impact on cognitive function [15], academic performance [16], and physical–mental health [17].

College students with early class start times obtain less sleep, relative to those with later class start times, due to earlier rise times [7, 18–21]. Delaying the start times of classes has been shown to result in increased sleep duration and better sleep quality [7, 22]. Since academic classes may not be the only type of activity that affects sleep timing [19, 23], it is important to know how other activities (e.g. exercise) impact the sleep timing and duration of college students. Furthermore, although the effect of class start time on sleep timing and duration has been investigated, particularly in middle and high school students [24–26], no study to date has examined how the total amount of time spent engaging in activities throughout the day and the timing of the last activity influence sleep timing and duration on a daily level among college students.

In the present study, we first describe the daily schedules and sleep–wake timing patterns in college students. We then investigate whether the timing, type, and documented total activity time of daily schedules affect the timing and duration of sleep. We hypothesized that (1) earlier schedule start times would be associated with earlier sleep onset times, earlier wake times, and shorter sleep duration; (2) later schedule end times would be associated with later sleep onset times, later wake times, and shorter sleep duration; and (3) increased daily documented total activity time (DDTAT) would be associated with later sleep onset times, earlier wake times, and shorter sleep duration.

## Materials and Methods

### Participants

Participants were 223 undergraduate college students attending the same university in the United States. Participants completed a screening questionnaire and were excluded if they were under 18 years or over 60 years old, did not use an Android phone, were pregnant, or had traveled more than one-time zone per week before the study and/or had plans of traveling at least one time zone away during the study. Eligible participants attended an information session about the study and provided informed consent prior to enrolling. Data collection were completed between 2013 and 2016; participants were studied for ~30 days in one of the six semesters. All research procedures were approved by the Committee on the Use of Humans as Experimental Subjects (COUHES) at the Massachusetts Institute of Technology in the United States and adhered to the Declaration of Helsinki. The study was registered on ClinicalTrials.gov under the identifier NCT02846077. Other outcomes derived from this protocol have been published previously [23, 27–29].

### Design

After providing informed consent, participants completed a brief pre-study questionnaire battery to collect information on demographics and the Morningness–Eveningness Questionnaire (MEQ) [30]. During the study, every day for approximately 30 consecutive days, participants wore an actigraphy device (MotionLogger-L, AMI, United States) and completed daily electronic-based surveys in the morning upon awakening and evening before bedtime. Data collection commenced within the first few weeks of the start of semester and ended before the start of the scheduled mid-semester break.

### Measures

#### Demographics.

Demographic factors including age, sex, academic year, and living situation were measured at baseline via self-report.

#### Sleep–wake timing.

Participants wore an actigraph on their nondominant wrist to monitor activity and ambient light information, and reported any instances of actigraph removal (i.e. when the monitor might get wet or damaged). Sleep onset time, wake time, and sleep duration (time between sleep onset and wake time minus wake after sleep onset) were determined from actigraphy analysis and corroborated with self-reported data from daily electronic morning surveys, as reported previously [23, 27].

#### College daily schedule.

**Figure 1.**
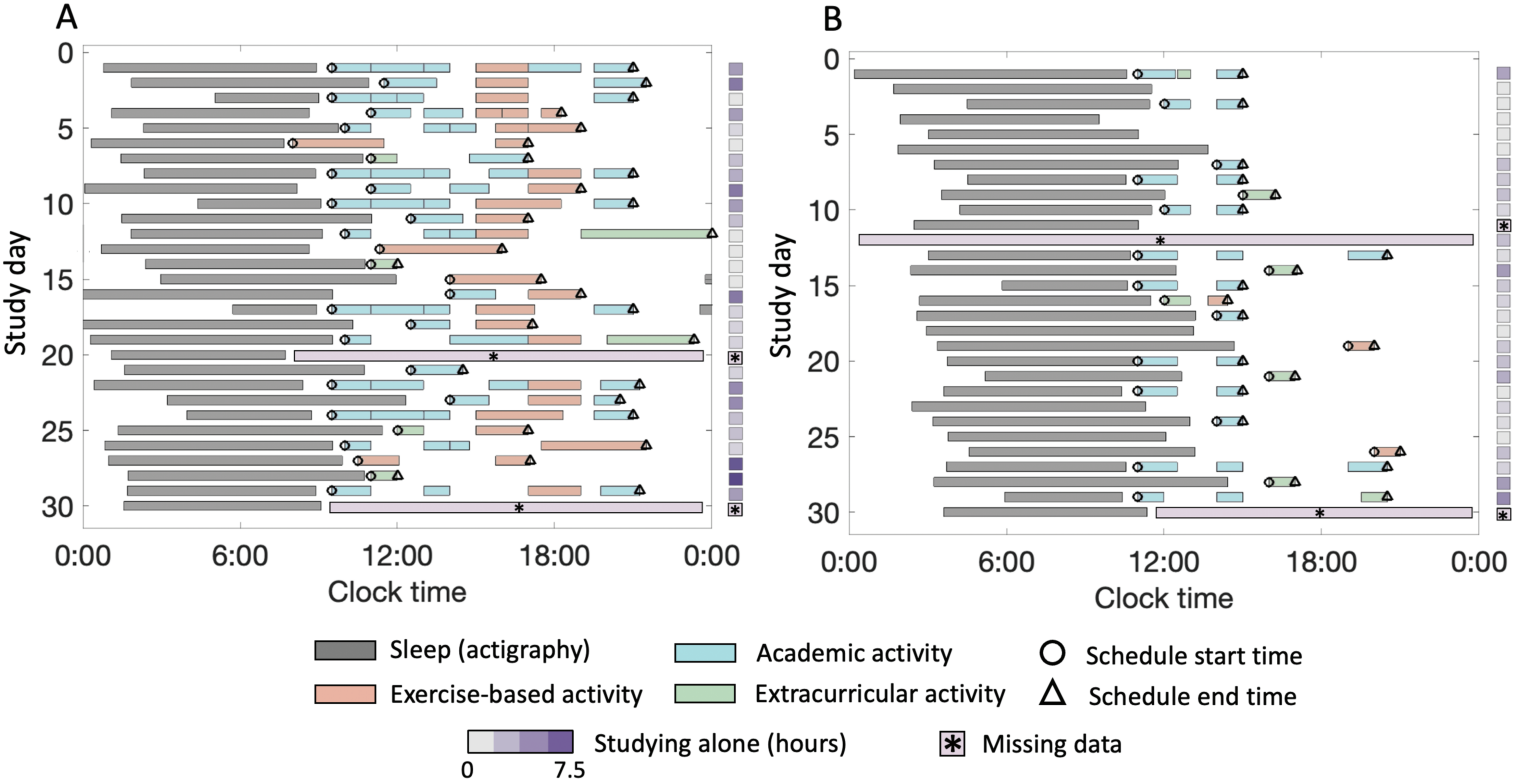
Raster plots of two participants, showing timing of sleep (from actigraphy and diaries) and wake-time attendance at academic, exercise-based, and extracurricular activities during the ~30 days of data collection. Duration spent studying alone is indicated by shades on the *y*-axis outside the plots, as the timing of studying alone was not collected. Circles and triangles indicate the start times of first activity and end times of last activity, respectively, on each day. A boxed asterisk indicates any missing data.

Participants completed an online survey each evening before bedtime regarding their attendance (yes/no response) at any academic (including classes, sections, seminars, labs, and study groups), exercise-based (including sports, gym, and cycling), and/or extracurricular (additional activities not otherwise covered by academic and exercise-based activities) activities that day. If participants responded that they attended an activity, they were asked to report the number of and the start time and duration (in minutes) of each occurrence of that activity. Participants also recorded the number of minutes they studied in total that day, not including any of the reported academic activities. DDTAT was calculated as the total time spent engaging in activities throughout the day, including academic, exercise-based, extracurricular, and time spent studying alone. Figure 1 shows examples of two college students’ daily schedules across the ~30-day monitoring interval.

#### Diurnal preference.

Diurnal preference was measured using the MEQ [30]. The MEQ is a 19-item self-assessment questionnaire that quantifies an individual’s preferred schedules for daily activities. Scores range between 16 and 86; lower scores indicate more eveningness preference and higher scores indicate more morningness preference.

#### Caffeine consumption and other substance use.

Participants responded via the daily evening survey to two dichotomous yes-no response questions of whether they (1) consumed caffeine or (2) used any other medications, drugs, or alcohol that day.

### Data analysis

All data analyses were performed using R Version v3.6.3. Descriptive statistics were calculated for daily schedule (timing, type, and documented total activity time) and sleep–wake timing variables. Paired *t*-tests assessed differences in daily schedules and sleep–wake timing patterns between weekdays and weekends, and between the first and last week of monitoring. Pearson correlations were used to determine whether the degree of weekday–weekend discrepancy in daily schedules was associated with the degree of weekday–weekend discrepancy in sleep–wake timing. Welch’s ANOVA [31] were employed to examine variations in daily schedule timing among different types of schedules, with the Games–Howell test [32] utilized as the post hoc analysis. Pearson correlations were used to explore associations between the MEQ and daily schedule variables.

**Figure 2.**
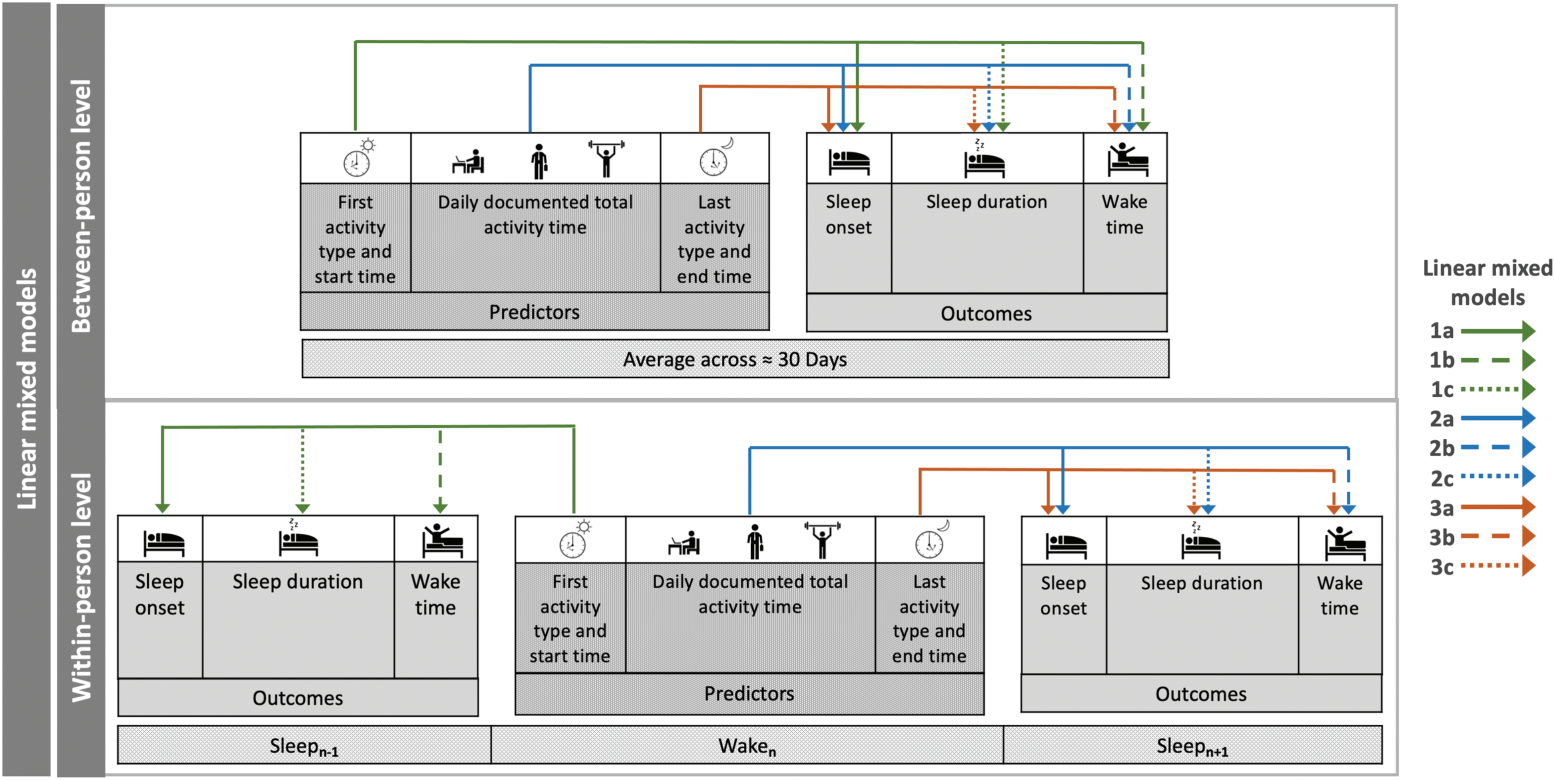
Schematic of the LMM associations tested between daily schedules and sleep variables at the between-person and within-person levels. Three LMMs (i.e. between-person and within-person effects of each daily schedule predictor) were fitted for each sleep outcome, totaling 9 LMMs. The dash type and color of the lines correspond to different LMMs (indicated by legend). The variable that is marked by the arrow refers to the outcome.

Linear mixed models (LMM; lme4 package, R) were used to analyze the effect of daily schedule timing, type, and documented total activity time on college students’ sleep onset time, wake time, and sleep duration. LMM analyses accounted for any potential day-to-day variability and the hierarchical nature of the nested data, i.e. the ~30 study days nested within each student. To determine the association between daily schedules and sleep–wake at both the between-person (average) and within-person (daily) levels, we calculated both the individual mean values across the ~30 study days (between-person effect), and daily raw deviations from an individual’s mean (within-person effect). We also conducted these analyses using median instead of mean; results were similar, so only mean-based analyses are reported below. Fixed effects included first activity start times, last activity end times, and DDTAT, with participants as random intercepts. Figure 2 illustrates the LMMs that were used for sleep outcomes (three LMMs for each sleep outcome), using restricted maximum likelihood estimation. Since the type of activity may influence students’ sleep–wake timing, we also accounted for the type of first and last activity at the within-person level and explored a stratified model of DDTAT (academic vs. studying alone vs. exercise-based vs. extracurricular). Satterthwaite’s approximation for degrees of freedom [33] was used to determine whether daily schedule predictors were statistically significantly associated with sleep–wake outcomes.

Prior to analyses, diagnostic checks for LMMs were performed via visual inspection and significance testing. Density plots and Q plots for residuals indicated mild non-normality and the homogeneity of variance assumption was reasonably met, with no clear trends in residuals. Two observations were removed due to being obvious entry errors, i.e. self-reported durations of studying alone (30 and 67 hours) exceeded the 24-hour day. To avoid confounding by daylight saving time transitions, 151 nights (2%) corresponding to clock transitions between daylight saving and standard time were excluded from analyses. To ensure robustness of the results against missing data, we performed a sensitivity analysis in which we repeated the primary analyses after excluding any participants with > 20% missing days (remaining sample: *N *= 172). Results of sensitivity analyses were similar to primary analyses with some minor differences (Supplementary Tables S1–S3). We analyzed the effect of study day on students’ daily schedules and sleep patterns throughout the monitoring period. The observed daily changes were very subtle (≤1 min per day, Supplementary Table S4). Study day was therefore omitted as a predictor from the primary models.

On many days in the dataset, the last event ended many hours before expected sleep onset time (49% of last events ended before 6 pm) and would therefore not be anticipated to have a strong influence on that night’s sleep timing. To investigate this, we performed secondary LMM analyses to test whether events that ended closer to the expected sleep onset time had a stronger influence on sleep. Specifically, we coded the last activity end time as the number of hours past a cutoff time of 10 pm (e.g. 11.30 pm coded as 1.5 hours), with a value of zero assigned for events that finished before the cutoff time (e.g. cutoff = 10 pm, with 5 pm coded as 0 hours). Supplemental LMM analyses were repeated for other cutoff times (6 pm, 7 pm, 8 pm and 9 pm, 11 pm, and 12 am; Supplementary Table S5).

All LMMs were adjusted for the covariates sex (female vs. male), academic year first (freshman) vs. second (sophomore) vs. third (junior) vs. fourth (senior) year, consumption of caffeine (yes vs. no) or any other medications, drugs, or alcohol (yes vs. no), and diurnal preference. Unadjusted models are included in Supplementary Tables S6–S8. We note that weekday vs. weekend (or school day vs. free day) was not included as a predictor in these models, since effects of school days vs. free days on sleep would be mediated by the event schedules themselves.

## Results

The sample comprised 223 undergraduate college students: 43% freshman, 20% sophomores, 14% juniors, and 23% seniors; ages 18–27 years; and 37% females. One hundred sixty-two (73%) students lived in a dormitory and 138 (62%) lived with roommates. All students reported attending academic activities and studying alone across the monitoring period; 23 (10%) students reported no exercise-based activities, 23 (10%) students reported no extracurricular activities, and 8 (4%) students reported neither exercise-based nor extracurricular activities.

### Schedules were earlier and busier on weekdays

**Figure 3.**
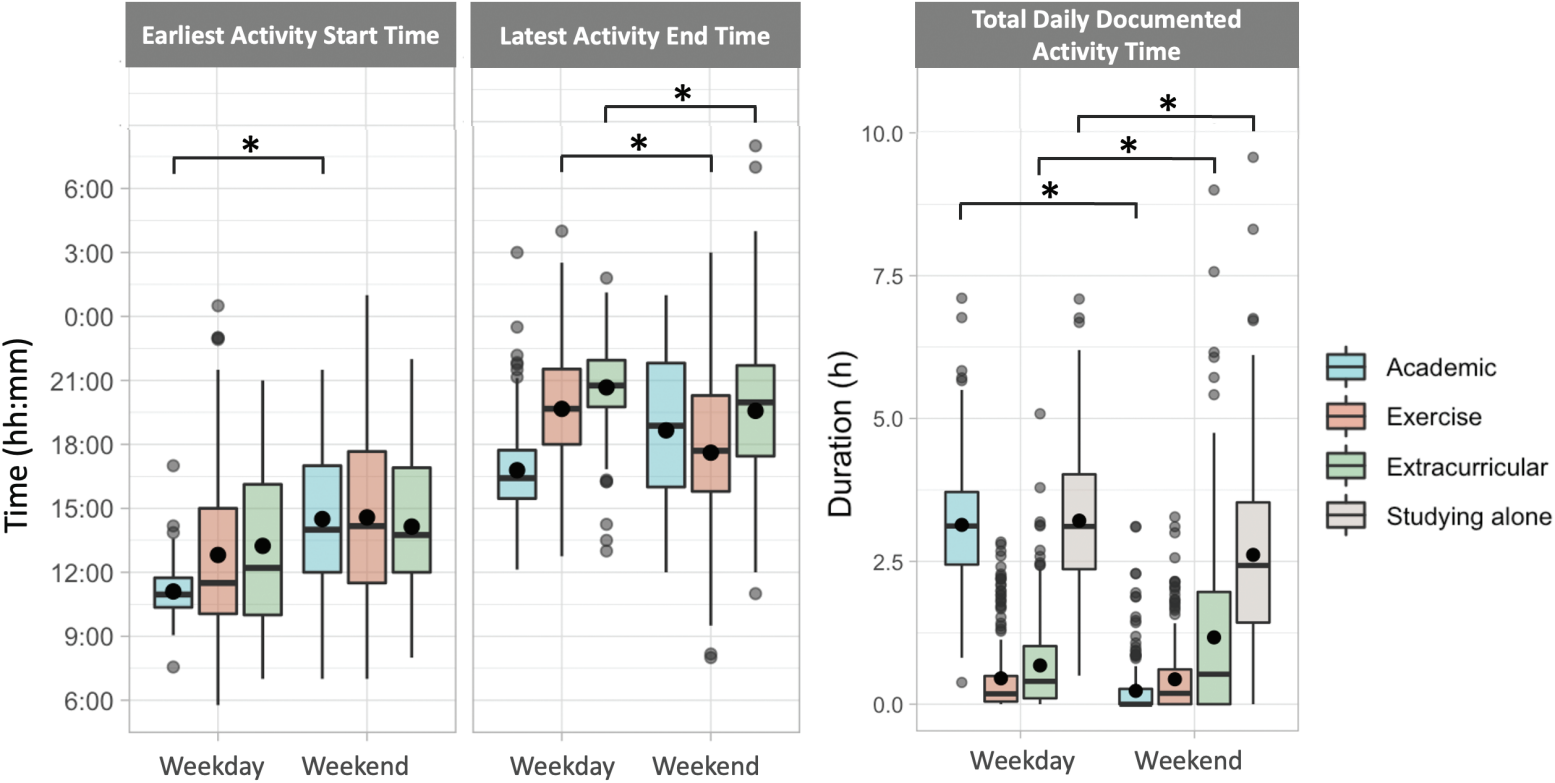
Boxplots of the average for each individual the scheduled start times of first event of the day, schedule end time of last event of the day, and the daily documented total activity time stratified by activity type and weekday versus weekend for the average value of each individual. Boxes indicate the first to third quartile; horizontal lines through the box indicate median; large black dots indicate mean; dots outside of boxes indicate outliers; vertical lines go from each quartile to the minimum or maximum. Note that the timing of studying alone was not collected, so is not included in the figure. Horizontal lines above the boxplots indicate significant weekday–weekend differences, where * signifies *p < *.0001.

**Figure 4.**
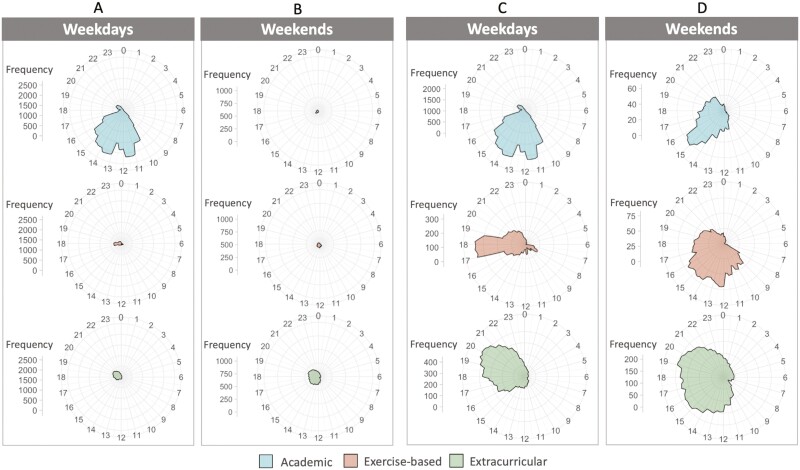
Polar plots showing the frequency distribution of activities by activity type (academic vs. exercise-based vs. extracurricular) for weekdays versus weekends across the approximate 30 study days of all participants. Frequency is plotted as a function of clock time (24 hours) around a wheel, with bands of the grid corresponding to the frequency of occurrence. Note that the frequency axes ranges are presented both on an equivalent scale (A and B) to facilitate a fair visual comparison between weekdays and weekends and zoomed-in scales (C and D) to better discern the temporal distributions.

The average timing and documented total activity time of college students’ daily schedules, stratified by type of activity and weekdays versus weekends, are described in Table 1 and illustrated in Figure 3. Figure 4 displays the frequency distribution of schedules at the daily level, stratified by activity type and weekdays versus weekends, over the approximate 30 study days. It can be inferred that weekdays are primarily characterized by academic responsibilities, whereas weekends by extracurricular activities. Schedule start times were significantly earlier on weekdays compared to weekends (11:07 ± 1:11 vs. 14:21 ± 3:03, *p < *.0001). On weekdays, students’ schedules started earlier if their first events were an academic activity (11:06 ± 1:04) compared to exercise-based (12:35 ± 4:13, *p < *.0001) or extracurricular activity (13:14 ± 3:45, *p < *.0001). Note that academic activities encompass a range of diverse academic engagements, extending beyond scheduled classes, including sections, seminars, labs, and study groups. During weekends, schedule start times were similar across activities.

**Table 1.** College Students’ Average Daily Schedule Stratified by Weekdays Versus Weekends

	Weekdays					Weekends					
	*N*	*Min*	*Max*	*M*	*SD*	*N*	*Min*	*Max*	*M*	*SD*	*p*
Schedule start time (hh:mm)	223	7:26	15:29	11:07	1:11	223	7:00	1:00^†^	14:21	3:03	<.0001
Academic	223	7:34	17:00	11:06	1:04	65	7:00	21:30	14:30	3:22	<.0001
Exercise-based	107	5:46	0:30^†^	12:35	4:13	133	7:00	1:00^†^	14:34	3:50	.075
Extracurricular	100	7:00	21:00	13:14	3:45	150	8:00	22:00	14:09	3:13	.568
Schedule end time (hh:mm)	223	12:22	23:47	18:07	1:54	223	8.17	7:00^†^	18:38	3:02	.170
Academic	221	12:08	3:00^†^	16:47	2:04	64	12:00	1:00^†^	18:40	3:26	.027
Exercise-based	159	12:45	4:00^†^	19:31	2:59	136	8:00	3:00^†^	17:37	3:42	<.0001
Extracurricular	178	13:00	1:48^†^	20:40	2:01	144	11:00	8:00^†^	19:34	3:23	<.0001
Daily documented total activity time (h)	223	3.4	11.9	7.5	1.9	223	0.0	11.4	4.4	2.3	<.0001
Academic	223	0.4	7.1	3.1	1.1	223	0.0	3.1	0.2	0.5	<.0001
Studying alone	223	0.5	7.1	3.2	1.3	223	0.0	9.6	2.6	1.6	<.0001
Exercise-based	223	0.0	2.8	0.5	0.6	223	0.0	3.3	0.4	0.6	.654
Extracurricular	223	0.0	5.1	0.7	0.8	223	0.0	9.0	1.2	1.5	<.0001
No activity	55	—	—	—	—	119	—	—	—	—	—

Note: N = number of participants; n = number of observations; Min = minimum; Max = maximum; M = mean; SD = standard deviation; † = time after midnight; p = probability of weekday-weekend difference at between-person level.

Schedule end times were not significantly different on weekdays compared to weekends (18:07 ± 1:54 vs. 18:38 ± 3:02, *p = *.17). On weekdays, schedules ended later if they ended with extracurricular activities (20:40 ± 2:01) or exercise-based activities (19:31 ± 2:59) compared to academic activities (16:47 *± *2:04, both *p < *.0001). On weekends, schedule end times differed by activity type: exercise-based (17:37 *± *3:42), academic (18:40 ± 3:26), and extracurricular (19:34 ± 3:23). Schedule start and end times showed greater variation on weekends compared to weekdays (*SD* = 3:03 and 3:02 hours on weekends vs. 1:11 and 1:54 hours on weekdays).

DDTAT was greater on weekdays compared to weekends (7.5 ± 1.9 hours vs. 4.4 ± 2.3 hours, *p < *.0001). On weekdays, students spent the most time studying (3.2 ± 1.3 hours) and academic activities (3.1 ± 1.1 hours), and less time in exercise (0.5 ± 0.6 hours) or extracurricular activities (0.7 ± 0.8 hours). On weekends, students continued to spend most of their time studying (2.6 *± *1.6 hours) but spent more time in extracurricular activities (1.2 ± 1.5 hours), similar time in exercise-based activities (0.4 ± 0.6 hours), and less time in academic activities (0.2 ± 0.5 hours).

Relative to the first week of monitoring, the last week showed an increase in time spent studying alone (2.8 ± 1.3 hours vs. 3.2 ± 1.6 hours, *p* < .01), and a decrease in time spent on exercise-based activities (0.5 ± 0.7 hours vs. 0.4 ± 0.7 hours, *p* < .01).

### Schedule irregularity was associated with sleep–wake irregularity between weekdays versus weekends

The degree of discrepancy in DDTAT between weekdays and weekends was positively correlated with the degree of discrepancy in sleep duration (*r* = 0.18, *p* < .01) and wake time (*r* = 0.21, *p* < .01) between these periods. In other words, individuals who had greater differences in their daily activity time between weekdays and weekends also had greater differences in their sleep duration and wake times. Furthermore, the degree of discrepancy in the weekday–weekend start times of individuals’ first scheduled activities was positively correlated with the degree of discrepancy in wake time (*r* = 0.29, *p* < .001). This implies that those with greater differences in the start time of their first scheduled activities between weekdays and weekends also tended to experience greater differences in their wake times. There was no significant association of the degree of discrepancy between weekdays versus weekends with the last scheduled activity end time or sleep onset.

### Schedule timing and busyness were associated with diurnal preference

**Figure 5.**
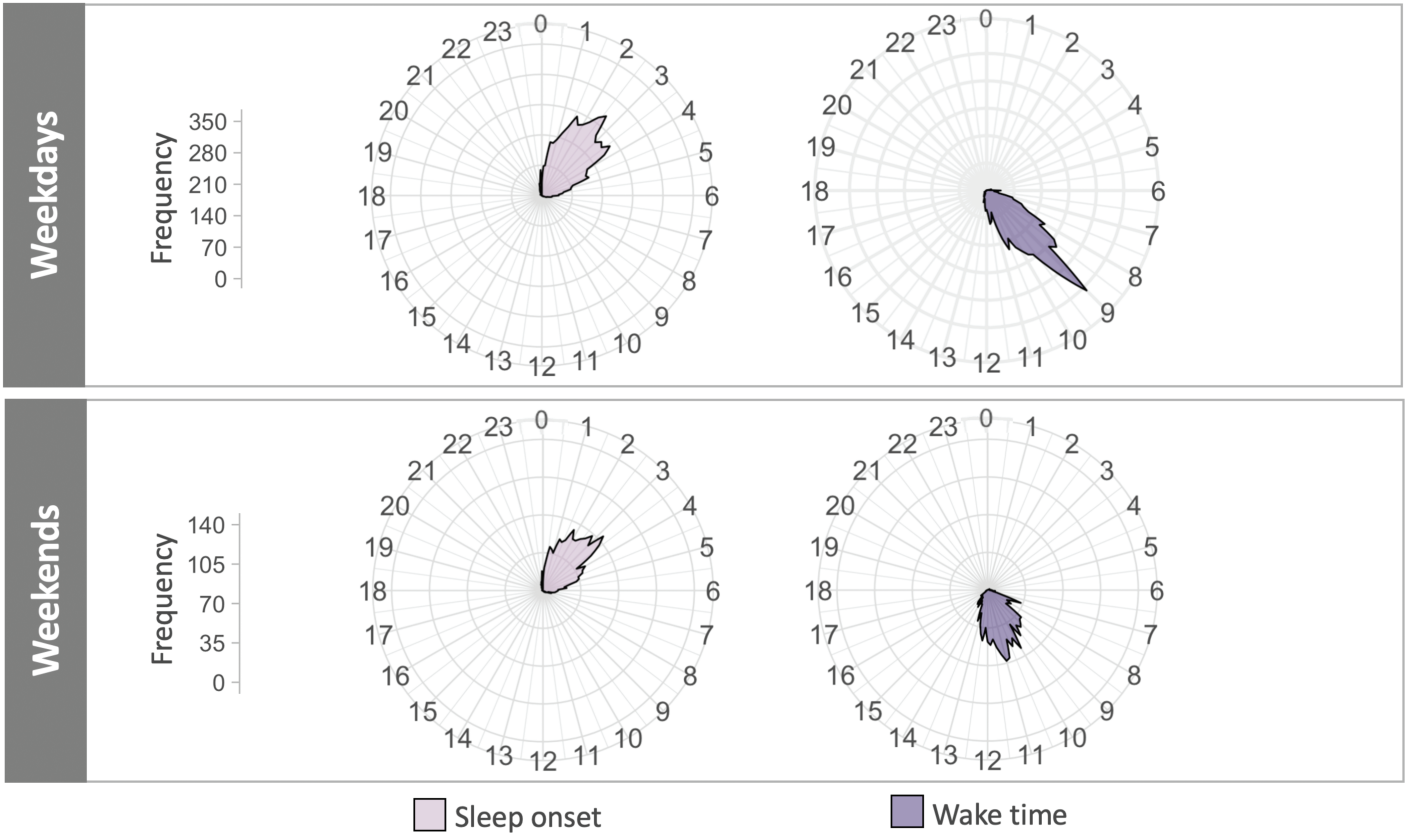
Polar plots showing the frequency distribution of sleep onset and wake times for weekdays versus weekends across the approximate 30 study days of all participants. Frequency is plotted as a function of clock time (24 hours) around a wheel, with bands of the grid corresponding to the frequency of occurrence. Note that the frequency axes ranges are presented both on an equivalent scale (A and B) to facilitate a fair visual comparison between weekdays and weekends.

An overview of actigraphy-based sleep–wake variables are presented in Table 2. Figure 5 also shows the frequency distribution of sleep onset and wake times on weekdays versus weekends at the daily level, across the approximate 30 study days. On weekdays, 152 (68%) students slept, on average, less than the recommended 7 to 9 hours per night for their age group [34]. The M ± SD MEQ score was 43.2 ± 9.2, including 13 (6%) morning types (scores: 59–86), 115 (52%) intermediate types (42–58), 94 (42%) evening types (16–41), and 1 missing. MEQ scores were negatively correlated with schedule start time (*r* = −0.25, *p* < .001) and positively correlated with DDTAT (*r* = 0.17, *p < *.05), meaning that morning and intermediate types tended to report earlier scheduled start time and increased DDTAT than evening types.

**Table 2.** College Students’ Average Daily Sleep–Wake Timing Stratified by Weekdays Versus Weekends

	Weekdays					Weekends					
	*N*	*Min*	*Max*	*M*	*SD*	*N*	*Min*	*Max*	*M*	*SD*	*p*
Sleep onset time (hh:mm)	223	23:43	5:53^†^	2:40^†^	1:10	223	23:25	7:04^†^	2:52^†^	1:19	<.0001
Wake time (hh:mm)	233	6:12	13:00	9:27	1:12	233	7:30	14:53	10:42	1:25	<.0001
Sleep duration (h)	223	4.3	9.0	6.7	0.8	223	4.5	9.6	7.0	1.0	<.0001

*N,* number of participants; *n,* number of observations; *M,* mean; *SD,* standard deviation; ^†^ = time after midnight; *p *= probability of weekday–weekend difference at between-person level. Sleep–wake timing and sleep duration are given as averages of weekday versus weekend obtained via actigraphy.

### Days with earlier start times are associated with curtailed sleep

Table 3 presents the results of LMMs testing the association between schedule start times and sleep outcomes, adjusting for covariates. At the between-person level (i.e. comparison of individuals’ schedule start times averaged across the ~30 study days), a 1-hour earlier first activity was associated with 26 minutes earlier sleep onset, 30 minutes earlier wake time, and no significant difference in sleep duration. At the within-person level (i.e. daily deviations from an individual’s average schedule start times), a 1 hour earlier first activity time was associated with a 4 minutes earlier sleep onset time on the preceding night, 16 minutes earlier wake time that day, and 11 minutes shorter sleep duration on the preceding night. Daily schedules starting with an exercise-based activity, relative to an extracurricular activity, were associated with a 16-minute earlier wake time that day.

**Table 3.** Results of LMMs Testing the Association Between Schedule Start Times and Sleep Outcomes, Adjusting for Covariates

	Sleep onset time		Wake time		Sleep duration	
Predictors	*β*	*SE*	*β*	*SE*	*β*	SE
Schedule start time (between)	0.43***	0.05	0.50***	0.04	0.06	0.05
Schedule start time (within)	0.07***	0.01	0.27***	0.01	0.19***	0.01
Academic first activity^†^ (within)	−0.10	0.08	−0.04	0.06	0.03	0.08
Exercise-based first activity^†^ (within)	−0.16	0.09	−0.27***	0.08	−0.17	0.10

Number of observations: 4091; groups: 222; ^†^denotes extracurricular first activity as the reference group; *β,* unstandardized coefficients in hours; SE, standard error; *p < .05, **p < .01, ***p < .001. Note that one LMM was fitted for each sleep–wake outcome.

### Events that end late are associated with curtailed sleep

Table 4 presents the results of LMMs testing the association between schedule end times and sleep outcomes, adjusting for covariates. At the between-person level, no significant difference was found between scheduled end time and sleep onset time, wake time or sleep duration. At the within-person level, a 1-hour later schedule end time was associated with a 2-minute later sleep onset time that night, 2 minutes shorter sleep duration, and no significant difference in next-day wake time. Daily schedules ending with an academic activity, relative to an extracurricular activity, were associated with an 8 minutes later sleep onset that night.

**Table 4.** Results of LMMs testing the association between schedule end times and sleep outcomes, adjusting for covariates

	Sleep onset time		Wake time		Sleep duration	
Predictors	*β*	SE	*β*	SE	*β*	SE
Schedule end time (between)	0.06	0.04	0.01	0.04	−0.04	0.03
Schedule end time (within)	0.04***	0.01	0.00	0.01	−0.04***	0.01
Academic last activity^†^ (within)	0.14*	0.06	0.03	0.06	−0.10	0.07
Exercise-based last activity^†^ (within)	0.03	0.07	0.07	0.07	0.04	0.08
Schedule end time h past 10 pm (between)	0.66***	0.19	0.24	0.18	−0.38*	0.16
Schedule end time h past 10 pm (within)	0.25***	0.03	0.14***	0.03	−0.10***	0.03

Number of observations: 4426; groups: 222; ^†^denotes extracurricular last activity was the reference group; *β*** =** unstandardized coefficients in hours; SE = standard error; *p < .05, **p < .01, ***p < .001. Note that two LMMs were fitted for each sleep–wake outcome.

Analysis of whether events extended into the night (i.e. the number of hours an event extended past 10 pm) revealed that at the between-person level, a 1-hour later schedule end time past 10 pm was associated with a 40-minute later sleep onset time and 23 minutes shorter sleep duration. At the within-person level, a 1-hour later schedule end time past 10 pm was associated with a 15-minute later sleep onset time that night, 6 minutes shorter sleep duration, and 8 minutes later next-day wake time.

### Busier schedules associated with curtailed sleep

Table 5 presents the results of LMMs exploring the association between DDTAT and sleep outcomes after adjusting for covariates. At the between-person level, a 1-hour increase in DDTAT was associated with a 6 minutes earlier wake time. At the within-person level, a 1-hour increase in DDTAT was associated with a 1-minute later sleep onset that night, 5 minutes earlier wake time the next day, and 5 minutes shorter sleep duration.

**Table 5.** Results of LMMs Testing the Association Between Daily Documented Total Activity Time and Sleep Outcomes After Adjusting for Covariates

	Sleep onset time		Wake time		Sleep duration	
Predictors	*β*	SE	*β*	SE	*β*	SE
Daily documented total activity time^†^ (between)	−0.03	0.04	−0.10**	0.04	−0.05	0.03
Daily documented total activity time^†^ (within)	0.02**	0.01	−0.08***	0.01	−0.09***	0.01
*Stratified daily documented total activity time*						
Academic (between)	−0.19*	0.08	−0.27***	0.05	−0.07	0.06
Studying alone (between)	0.13*	0.05	0.05	0.02	−0.05	0.04
Exercise-based (between)	−0.23*	0.11	−0.27**	0.10	−0.01	0.09
Extracurricular (between)	−0.08	0.07	−0.17*	0.07	−0.06	0.06
Academic (within)	−0.02	0.01	−0.08***	0.01	−0.06***	0.01
Studying alone (within)	0.05***	0.01	−0.09***	0.01	−0.13***	0.01
Exercise-based (within)	−0.02	0.03	−0.07**	0.03	−0.05	0.03
Extracurricular (within)	0.02	0.01	−0.02	0.01	−0.03*	0.01

Number of observations: 5238; groups: 222; Daily documented total activity time^†^ was calculated as the total time spent engaging in academic, studying alone, exercise-based, and extracurricular activities; *β* = unstandardized coefficients in hours; SE = standard error; *p < .05, **p < .01, ***p < .001. Note that two LMMs (non-stratified vs. stratified by activity) were fitted for each sleep–wake outcome.

Stratifying DDTAT by activity type at the between-person level, a 1-hour increase in academic activities was associated with 11 minutes earlier sleep onset time and 16 minutes earlier wake time. A 1-hour increase in time spent studying alone was associated with 8 minutes later sleep onset time. A 1-hour increase in exercise-based activities was associated with 14 minutes earlier sleep onset time and 15 minutes earlier wake time. A 1-hour increase in extracurricular activities was associated with 10 minutes earlier wake time. At the within-person level, a 1 hour increase in academic activities was associated with 5 minutes earlier next-day wake time, and 4 minutes shorter sleep duration. A 1-hour increase in time spent studying alone was associated with 3 minutes later sleep onset time that night, 5 minutes earlier next-day wake time, and 8 minutes shorter sleep duration. A 1-hour increase in exercise-based activities was associated with 4 minutes earlier next-day wake time, and a 1-hour increase in extracurricular activities was associated with 2 minutes shorter sleep duration.

## Discussion

The current study investigated the organization of sleep–wake patterns relative to daily schedules in college students across a month at one school. By examining the timing, type, and total activity time of daily schedules, we were able to dissect how different components of daily schedules influence sleep at both the within-person and between-person levels. Our hypothesis that earlier schedule start times would be associated with earlier sleep onset times and earlier wake times was supported at the within- and between-person levels. However, sleep duration was only impacted by earlier scheduled start times at the within-person level, likely due to the effect of earlier start times being greater on wake times than sleep onset times. Later schedule end times were associated with later sleep onset times and shorter sleep duration at the within-person level; effects were stronger when looking at days where schedule end times extended into the night. As anticipated, increased DDTAT were associated with later sleep onset and shorter sleep duration at the within-person level, and earlier wake time at the within- and between-person levels.

Our findings demonstrate how scheduled start times of college students may shape their sleep patterns. Studies have shown that students with later class or lecture start times, relative to those with early class start times, receive more sleep due to later rise times [7, 18–21]. However, academic classes may not be the first or only planned activity that affects sleep–wake timing [19, 23]. By taking recorded exercise and extracurricular activities into account, our results provide a more complete picture. As expected, we found that within individuals, schedule start times had a substantial effect on wake times, with modest changes in sleep onset time. Specifically, having a schedule that started earlier in the day was associated with earlier sleep and wake times relative to their usual schedule timing, but with a net shorter sleep duration, due to a larger advance in wake time than sleep onset time. These findings suggest that students may be exerting little effort and/or have minimal ability to initiate sleep earlier than usual in anticipation of an early start the following morning, resulting in shorter sleep duration. Difficulties initiating sleep earlier than usual may be due to attempting to sleep at an adverse circadian phase, i.e. the wake maintenance zone, where wakefulness is strongly promoted by the circadian clock [35, 36]. Furthermore, having a schedule that started later in the day resulted in later sleep and wake times relative to their usual schedule timing, but with a net longer sleep duration due to a larger delay in wake time than sleep onset time. These findings are similar to results from middle and high school students: delaying school start times generally increases sleep duration [24–26], with modeling showing that the extent of this benefit depends on how early school starts relative to sunrise [37]. It should be noted that the magnitude of net increase in sleep duration observed with delayed schedule start times in our study was less pronounced than what has been reported among middle and high school students [26]. This variance may be attributed to our sample having later scheduled start times, on average, compared to typical high school students in contexts such as the United States, where average start times can be as early as 8 am [38, 39]. Nonetheless, despite the relatively modest impact of schedule start time on sleep duration in our study, it is important to recognize that the gradual accumulation of sleep deficit can still potentially lead to chronic sleep deprivation, which can in turn impact both health [40] and neurobehavioral functions [41]. When looking across individuals, we found that students who had later schedule start times on average had later sleep and wake times, but no difference in sleep duration. This may reflect the control college students have over the timing of their schedules, where students with later habitual sleep timing may be inclined to self-select start times that align with their more delayed sleep schedule.

On days when schedules ended later than usual, students went to sleep later that night and had shorter sleep duration. This effect was stronger when examining the degree to which end times extended into the night past 10 pm. Within individuals, we found that every 1 hour that an event extended beyond 10 pm meant sleep onset occurred 15 minutes later than normal, which was partially compensated by waking 8 minutes later the following day. This suggests that students were more willing to schedule late events or stay up later when there may have been an opportunity to sleep in the following day. Looking between individuals, we found that every 1 hour that the average schedule end time extended beyond 10 pm meant that sleep onset occurred 40 minutes later and sleep duration was 23 minutes shorter. This finding may hold significant clinical implications, as prior research has shown that losing just 16 minutes of sleep in a single night is associated with increased cognitive interference, such as off-task and distracting thoughts [42]. Our finding also suggests that individuals with more delayed schedules on average consistently accrue sleep loss. This may occur due to the conflict of very delayed wake times with events scheduled early on certain days such as classes. This pattern is common in (counterclockwise-rotating) shift work (e.g. nursing) whereby the late end time of an evening shift and the early start time of the subsequent day shift restrict the total time available for sleep, which can have functional consequences such as increased sleepiness and decreased performance [43, 44].

Different activity types had differential effects on sleep and wake. We found that schedules starting with an exercise-based activity (e.g. sports, gym, and cycling) were associated with earlier wake times that day, compared to days starting with an extracurricular activity (i.e. activities not otherwise covered by academic and exercise-based activities). This could potentially be due to exercise start times (12:35 ± 4:13) (see also range of exercise start times in Figure 3) typically being earlier than extracurricular activities (13:14 ± 3:45), as indicated in this sample on weekdays. While the timing of exercise could potentially be influenced by diurnal preference, there was no significant correlation between diurnal preference and the average timing of exercise as the first scheduled activity. This could be reflective of sports teams having practices in the morning, which are not scheduled by students. We also found that daily schedules ending with an academic activity were associated with later sleep onset that night, relative to daily schedules ending with an extracurricular-based activity. This may be due to stress or other factors related to these academic activities [45], leading to presleep arousal at bedtime [46].

College students experience highly variable levels of DDTATs (5th–95th percentile range: 3.7–9.6 hours). We found that on days when DDTATs were greater than usual, students went to sleep later that night, woke up earlier the next day, and had shortened sleep duration, although these effects were small. These results have two plausible interpretations. First, an increased DDTAT may place competing demands on time, resulting in sleep duration being sacrificed. Second, an increased DDTAT may reflect periods of higher workload and stress, resulting in greater restlessness at bedtime and consequently impaired sleep [47].

The effect of DDTATs on sleep–wake patterns, albeit modest, varied by activity type. Days with increased time spent on academic activities were associated with an earlier next-day wake time and shorter sleep duration. Increased time spent studying alone was associated with later sleep onset time that night, earlier next-day wake time, and shorter sleep duration. Of note, the timing of data collection was designed to not be during major mid-term exams; students may still have had other exams that would be expected to be associated with later academic activities and less sleep the night before the exam. These findings also may be due to mental exhaustion or work immersion interfering with sleep patterns. Increased time spent on exercise-based activities was associated with earlier sleep onset time at the between-person level. This finding may be related to how exercise raises core body temperature and leads to a subsequent cooling down period that helps the body achieve natural readiness for sleep, resulting in earlier sleep onset times [48]. This process also likely impacts the distal-proximal skin temperature gradient, which is a significant predictor for faster sleep onset latency [49]. However, this increase in exercise time did not correspond to significant change in sleep duration, since increased time spent on exercise was also associated with earlier wake times at the between-person level. This finding differs from a previous study where moderate-vigorous physical activity in college students was associated with earlier sleep onset time and longer sleep duration [50]. The difference in results may be due to our different methods of measuring exercise-based activity, where we relied on self-report daily diaries, whereas the previous study used actigraphic physical activity measures. Our findings of earlier wake times offset any potential extension in sleep duration, resulting in an overall neutral effect. Increased time spent on extracurricular activities was associated with shorter sleep duration at the within-person level and earlier wake time at the between-person level. This finding aligns with previous work showing that those who maintain a part-time job during college wake up earlier and sleep less in comparison to non-working students [51]. Overall, an increased DDTAT often results in a reduced opportunity for sleep in college students. Future research may explore robust daily schedules in combination with data describing students’ abilities to manage their time effectively, which have been shown in previous studies to be a significant predictor of sleep health in college students [52].

In our study, we generally observed a modest influence of the daily schedule on sleep duration, but a stronger relationship of daily schedules with sleep–wake timing. Previous research consistently emphasizes the importance of obtaining adequate sleep duration, as insufficient sleep has been linked to a range of adverse cognitive [53] and metabolic [54] consequences. However, it is crucial to also recognize the importance of sleep timing and regularity. Individuals with irregular sleep patterns, when compared to those maintaining consistent sleep routines, display poorer academic performance, even when sleep durations are comparable [1]. Furthermore, greater sleep–wake irregularity has been associated with poorer health outcomes, such as greater body mass index [55], increased depression severity and perceived stress, as well as higher cardiometabolic risk [56]. This highlights the necessity of considering not only the quantity of sleep but also its consistency and timing for optimal well-being and functioning.

A strength of this study is that our research design enabled a detailed exploration of how a range of daily events impact sleep timing at the intra- and inter-individual levels. This enabled us to account for variation in sleep timing both between, and within, individuals, and extends beyond the focus on academic classes/lectures and sleep. Nevertheless, this study had some important limitations. First, participants were recruited from a single Massachusetts university in the United States, which may reduce generalizability: students from other universities may have different schedules. Second, students’ daily schedules were based on self-reports and were not corroborated with their college timetables or calendars. Third, there was a higher proportion of male participants in our sample (63%), which could reduce the generalizability of our results to the entire college student population. However, using sex as covariate did not significantly impact any of our main LMM findings. Fourth, the timing of studying alone was not measured; it is therefore possible that students may have started or ended their daily schedule with studying. Fifth, other activities, such as spontaneous social and non-documented activities (internet browsing, social media, etc.), were not included in analyses; these would also be expected to affect sleep timing and quantity. Lastly, our study did not explore other sleep characteristics, such as perceived sleep quality.

In conclusion, this study offered novel insights into the organization of sleep–wake patterns around daily schedules in one set of college students. By looking beyond academic scheduling, and taking exercise and extracurricular activities into consideration, our study provided a more holistic view of how college students’ daily schedules can influence sleep–wake timing, which may pose daily challenges to good sleep hygiene and sleep–wake regularity. Acute changes in daily schedules can induce corresponding changes in sleep–wake timing. For instance, earlier start times than normal induce shorter sleep duration. Future research may build upon these findings to promote time management skills in students, which may buffer against sleep loss during busy times in a typical semester. Although these findings are specific to one university’s college students, it is possible these findings may be generalizable to other college students, industries, and behavioral contexts that involve scheduling activities. In a college context, institutions could consider offering more class timetabling opportunities to accommodate differing schedules.

## Supplementary Material

Supplementary material is available at *SLEEP* online.

zsad278_suppl_Supplementary_Tables_S1-S8

## Data Availability

Detailed data access will require institutional review board approval and a data use agreement with Mass General Brigham. Further information and requests for data should be directed to and will be fulfilled by Dr. Elizabeth B. Klerman (ebklerman@hms.harvard.edu).
