# Supplementary material for: The organization of sleep–wake patterns around daily schedules in college students
Source: Sleep. 2023 Nov 1;47(9):zsad278. doi: 10.1093/sleep/zsad278 (PMC11381563; doi:10.1093/sleep/zsad278)
Supplement: zsad278_suppl_Supplementary_Tables_S1-S8 [file zsad278_suppl_supplementary_tables_s1-s8.docx]

# The organization of sleep-wake patterns around daily schedules in college students

**Authors:**

Sinh Lu^1^, Julia E. Stone^1^, Elizabeth B. Klerman^2,3,4^, Andrew W. McHill^3,4,5^, Laura K. Barger^3,4^, Rebecca Robbins^3,4^, Dorothee Fischer^6^, Akane Sano^7,8^, Charles A. Czeisler^3,4^, Shantha M. W. Rajaratnam^1,3,4^, Andrew J. K. Phillips^1,3,4^

**Author affiliations:**

*^1^Turner Institute for Brain and Mental Health, School of Psychological Sciences, Monash University, Clayton, Victoria, Australia*

*^2^Department of Neurology, Massachusetts General Hospital, Boston, Massachusetts, USA*

*^3^Division of Sleep and Circadian Disorders, Departments of Medicine and Neurology, Brigham and Women’s Hospital, Boston, Massachusetts, USA*

*^4^Division of Sleep Medicine, Harvard Medical School, Boston, Massachusetts, USA*

*^5^Sleep, Chronobiology, and Health Laboratory, School of Nursing, Oregon Health & Science University, Portland, Oregon, USA*

*^6^Department of Sleep and Human Factors Research, Institute for Aerospace Medicine, German Aerospace Center, Cologne, Germany*

*^7^Department of Electrical and Computer Engineering, Rice University, Houston, Texas, USA*

*^8^Affective Computing Group, Media Lab, Massachusetts Institute of Technology, Cambridge, MA, USA*

# Corresponding author: Dr. Andrew J. K. Phillips, [andrew.philips@monash.edu](mailto:andrew.philips@monash.edu)

# Supplementary Material

**Tables S1-3.** Sensitivity analyses to test robustness of primary results against missing data.

**Table S4.** Secondary analyses to test the impact of study day on students' daily schedule and sleep patterns throughout the monitoring period.

**Table S5.** Secondary analyses to test whether events that extended into the night (cut-off times: 6pm, 7pm, 8pm, 9pm, 11pm and 12am) had a stronger influence on sleep.

**Table S6-8.** Unadjusted analyses testing associations between daily schedule predictors and sleep-wake outcomes.

# Table S1

| **Table S1**. Results of LMMs testing the association between schedule start times and sleep outcomes, adjusting for covariates and removing participants with >20% missing days (*N* = 51) | | | | | | |
| --- | --- | --- | --- | --- | --- | --- |
|  | Sleep onset time | | Wake time | | Sleep duration | |
| **Predictors** | **β** | **SE** | **β** | **SE** | **β** | **SE** |
| Schedule start time (between) | 0.38*** | 0.06 | 0.51*** | 0.05 | 0.13* | 0.06 |
| Schedule start time (within) | 0.06*** | 0.01 | 0.27*** | 0.01 | 0.19*** | 0.01 |
| Academic first activity^†^ (within) | −0.17* | 0.08 | −0.11 | 0.06 | 0.04 | 0.08 |
| Exercise-based first activity^†^ (within) | −0.16 | 0.10 | −0.29*** | 0.08 | −0.17 | 0.10 |
| Number of observations: 3529; Groups: 171; ^†^ denotes extracurricular first activity as the reference group; β **=** unstandardized coefficients in hours; SE = standard error; **p* < .05, ***p* < .01, ****p* < .001. | | | | | | |

# Table S2

| **Table S2*.*** Results of LMMs testing the association between schedule end times and sleep outcomes, adjusting for covariates and removing participants with >20% missing days (*N* = 51) | | | | | | |  |
| --- | --- | --- | --- | --- | --- | --- | --- |
|  | Sleep onset time | | Wake time | | Sleep duration | | |
| **Predictors** | **β** | **SE** | **β** | **SE** | **β** | **SE** | |
| Schedule end time (between) | 0.04 | 0.04 | 0.01 | 0.04 | −0.01 | 0.04 | |
| Schedule end time (within) | 0.04*** | 0.01 | <−0.01 | 0.01 | −0.04*** | 0.01 | |
| Academic last activity^†^ (within) | 0.13 | 0.07 | 0.02 | 0.07 | −0.09 | 0.07 | |
| Exercise-based last activity^†^ (within) | −0.02 | 0.07 | 0.03 | 0.07 | 0.05 | 0.08 | |
| Schedule end time _h past 10pm_ (between) | 0.42* | 0.20 | 0.05 | 0.20 | −0.32 | 0.18 | |
| Schedule end time _h past 10pm_ (within) | 0.23*** | 0.03 | 0.12*** | 0.03 | −0.10** | 0.03 | |
| Number of observations: 3743; Groups: 171; ^†^ denotes extracurricular last activity was the reference group; β **=** unstandardized coefficients in hours; SE = standard error; **p* < .05, ***p* < .01, ****p* < .001. | | | | | | |  |

# Table S3

| **Table S3.** Results of LMMs testing the association between daily documented total activity time and sleep outcomes after adjusting for covariates and removing participants with >20% missing days (*N* = 51) | | | | | | | |  |
| --- | --- | --- | --- | --- | --- | --- | --- | --- |
|  | | Sleep onset time | | Wake time | | Sleep duration | | |
| **Predictors** | | **β** | **SE** | **β** | **SE** | **β** | **SE** | |
| Daily documented total activity time^†^ (between) | | −0.03 | 0.04 | −0.08 | 0.04 | −0.05 | 0.03 | |
| Daily documented total activity time^†^ (within) | | 0.02** | 0.01 | −0.08*** | 0.01 | −0.09*** | 0.01 | |
| Stratified daily documented total activity time | |  |  |  |  |  |  | |
| Academic (between) | −0.23** | 0.09 | −0.29*** | 0.08 | −0.05 | 0.08 | |  |
| Studying alone (between) | 0.12* | 0.05 | 0.08 | 0.05 | −0.03 | 0.05 | |  |
| Exercise-based (between) | −0.11 | 0.11 | −0.11 | 0.10 | 0.01 | 0.09 | |  |
| Extracurricular (between) | −0.02 | 0.08 | −0.16* | 0.08 | −0.11 | 0.07 | |  |
| Academic (within) | −0.01 | 0.01 | −0.08*** | 0.01 | −0.06*** | 0.01 | |  |
| Studying alone (within) | 0.05*** | 0.01 | −0.09*** | 0.01 | −0.12*** | 0.01 | |  |
| Exercise-based (within) | −0.02 | 0.03 | −0.09** | 0.03 | −0.06 | 0.03 | |  |
| Extracurricular (within) | 0.02 | 0.01 | −0.03 | 0.01 | −0.04* | 0.02 | |  |
| Number of observations: 4378; Groups: 171; Daily documented total activity time^†^ was calculated as the total time spent engaging in academic, studying alone, exercise-based, and extracurricular activities; β **=** unstandardized coefficients in hours; SE = standard error; **p* < .05, ***p* < .01, ****p* < .001. | | | | | | | |  |

# Table S4

**Table S4.** Results of LMMs testing the impact of study day on students' daily schedule and sleep patterns throughout the monitoring period

|  | |  | | **Predictor**: Study day | |
| --- | --- | --- | --- | --- | --- |
|  | |  | | **β** | **SE** |
| Schedule timing | | |  |  |  |
| Schedule start time | |  | <0.01 | <0.01 |  |
| Schedule end time | |  | −0.02* | 0.01 |  |
| Daily documented total activity time | | |  |  |  |
| Academic | |  | <−0.01 | <0.01 |  |
| Studying alone | |  | 0.02*** | <0.01 |  |
| Exercise-based | |  | <−0.01 | <0.01 |  |
| Extracurricular | |  | <−0.01 | <0.01 |  |
| Sleep/wake timing | | |  |  |  |
| Sleep onset time | |  | 0.01* | <0.01 |  |
| Wake time | |  | 0.01* | <0.01 |  |
| Sleep duration | |  | <−0.01 | <0.01 |  |
| β **=** unstandardized coefficients in hours; SE = standard error; **p* < .05, ***p* < .01, ****p* < .001. | | | | | |

# Table S5

| **Table S5*.*** Results of LMMs testing the association between schedule end times and sleep outcomes, adjusting for covariates | | | | | | |  |
| --- | --- | --- | --- | --- | --- | --- | --- |
|  | Sleep onset time | | Wake time | | Sleep duration | | |
| **Predictors** | **β** | **SE** | **β** | **SE** | **β** | **SE** | |
| Schedule end time _h past 6pm_ (between) | 0.11 | 0.06 | 0.02 | 0.06 | −0.07 | 0.05 | |
| Schedule end time _h past 6pm_ (within) | 0.07*** | 0.01 | 0.02 | 0.01 | −0.05*** | 0.01 | |
| Schedule end time _h past 7pm_ (between) | 0.17* | 0.08 | 0.04 | 0.08 | −0.10 | 0.06 | |
| Schedule end time _h past 7pm_ (within) | 0.09*** | 0.01 | 0.03** | 0.01 | −0.05*** | 0.01 | |
| Schedule end time _h past 8pm_ (between) | 0.25** | 0.10 | 0.07 | 0.10 | −0.15 | 0.08 | |
| Schedule end time _h past 8pm_ (within) | 0.13*** | 0.02 | 0.06*** | 0.02 | −0.06*** | 0.02 | |
| Schedule end time _h past 9pm_ (between) | 0.40** | 0.13 | 0.12 | 0.13 | −0.25* | 0.11 | |
| Schedule end time _h past 9pm_ (within) | 0.18*** | 0.02 | 0.09*** | 0.02 | −0.07*** | 0.02 | |
| Schedule end time _h past 11pm_ (between) | 0.98*** | 0.26 | 0.45 | 0.25 | −0.49* | 0.22 | |
| Schedule end time _h past 11pm_ (within) | 0.25*** | 0.03 | 0.14*** | 0.03 | −0.09** | 0.03 | |
| Schedule end time _h past 12am_ (between) | 1.40*** | 0.37 | 0.80* | 0.36 | −0.59 | 0.31 | |
| Schedule end time _h past 12am_ (within) | 0.25*** | 0.03 | 0.14*** | 0.03 | −0.09** | 0.03 | |
| Number of observations: 4426; Groups: 222; β **=** unstandardized coefficients in hours; SE = standard error; **p* < .05, ***p* < .01, ****p* < .001. | | | | | | |  |

# Table S6

| **Table S6.** Results of LMMs testing the association between schedule start times and sleep outcomes | | | | | | |
| --- | --- | --- | --- | --- | --- | --- |
|  | Sleep onset time | | Wake time | | Sleep duration | |
| **Predictors** | **β** | **SE** | **β** | **SE** | **β** | **SE** |
| Schedule start time (between) | 0.53*** | 0.06 | 0.61*** | 0.05 | 0.06 | 0.04 |
| Schedule start time (within) | 0.07*** | 0.01 | 0.27*** | 0.01 | 0.18*** | 0.01 |
| Academic first activity^†^ (within) | −0.14 | 0.07 | −0.10 | 0.06 | 0.02 | 0.07 |
| Exercise-based first activity^†^ (within) | −0.21* | 0.09 | −0.32*** | 0.07 | −0.17 | 0.09 |
| Number of observations: 4470; Groups: 223; ^†^ denotes extracurricular first activity as reference for comparison; β **=** unstandardized coefficients in hours; SE = standard error; **p* < .05, ***p* < .01, ****p* < .001. | | | | | | |

# Table S7

| **Table S7*.*** Results of LMMs testing the association between schedule end times and sleep outcomes | | | | | | | |
| --- | --- | --- | --- | --- | --- | --- | --- |
|  | Sleep onset time | | Wake time | | Sleep duration | | |
| **Predictors** | **β** | **SE** | **β** | **SE** | **β** | **SE** | |
| Schedule end time (between) | 0.04 | 0.04 | 0.01 | 0.04 | −0.02 | 0.03 | |
| Schedule end time (within) | 0.04*** | 0.01 | *<*−0.01 | 0.01 | −0.04*** | 0.01 | |
| Academic last activity^†^ (within) | 0.12* | 0.06 | 0.01 | 0.06 | −0.10 | 0.07 | |
| Exercise-based last activity^†^ (within) | 0.02 | 0.07 | 0.06 | 0.07 | 0.04 | 0.07 | |
| Schedule end time _h past 10pm_ (between) | 0.76*** | 0.22 | 0.41 | 0.23 | −0.31* | 0.15 | |
| Schedule end time _h past 10pm_ (within) | 0.25*** | 0.03 | 0.14*** | 0.03 | −0.09*** | 0.03 | |
| Number of observations: 4500; Groups: 223; ^†^ denotes extracurricular first activity as reference for comparison; β **=** unstandardized coefficients in hours; SE = standard error; **p* < .05, ***p* < .01, ****p* < .001. | | | | | | |  |

# Table S8

| **Table S8.** Results of LMMs testing the association between daily documented total activity time and sleep outcomes | | | | | | | |  |
| --- | --- | --- | --- | --- | --- | --- | --- | --- |
|  | | Sleep onset time | | Wake time | | Sleep duration | | |
| **Predictors** | | **β** | **SE** | **β** | **SE** | **β** | **SE** | |
| Daily documented total activity time^†^ (between) | | −0.12** | 0.04 | −0.19*** | 0.04 | −0.05 | 0.03 | |
| Daily documented total activity time^†^ (within) | | 0.02** | 0.01 | −0.08*** | 0.01 | −0.09*** | 0.01 | |
| Stratified daily documented total activity time | |  |  |  |  |  |  | |
| Academic (between) | −0.26** | 0.09 | −0.35*** | 0.09 | −0.07 | 0.06 | |  |
| Studying alone (between) | 0.06 | 0.06 | <−0.01 | 0.06 | −0.04 | 0.04 | |  |
| Exercise-based (between) | −0.36** | 0.12 | −0.40** | 0.12 | −0.01 | 0.08 | |  |
| Extracurricular (between) | −0.17 | 0.08 | −0.24** | 0.08 | −0.03 | 0.06 | |  |
| Academic (within) | −0.02 | 0.01 | −0.09*** | 0.01 | −0.06*** | 0.01 | |  |
| Studying alone (within) | 0.05*** | 0.01 | −0.10*** | 0.01 | −0.14*** | 0.01 | |  |
| Exercise-based (within) | −0.02 | 0.03 | −0.07** | 0.03 | −0.05 | 0.03 | |  |
| Extracurricular (within) | 0.02 | 0.01 | −0.02 | 0.01 | −0.04* | 0.01 | |  |
| Number of observations: 5333; Groups: 223; Daily documented total activity time^†^ was calculated as the total time spent engaging in academic, studying alone, exercise-based, and extracurricular activities; β **=** unstandardized coefficients in hours; SE = standard error; **p* < .05, ***p* < .01, ****p* < .001. | | | | | | | |  |
